# Effect of Indenter Load on Vickers Microhardness and Indentation Depth of One Resin Composite

**DOI:** 10.3390/ma17246156

**Published:** 2024-12-17

**Authors:** Richard B. Price, Braden Sullivan

**Affiliations:** Department Dental Clinical Sciences, Dalhousie University, 5981 University Avenue, Halifax, NS B3H 4R2, Canada; braden.sullivan@dal.ca

**Keywords:** composite resin, polymerization, light-curing, hardness measurement, monomer conversion, indentation size effect

## Abstract

The load and size of the indentation may affect the hardness value. This study investigated the effect of the indentation size on the microhardness of one resin-based composite (RBC). Metal molds 4 mm deep and 12 mm in diameter were filled with Tetric EvoCeram Bulk Fill (Ivoclar) and light-cured for 10 s using a broad-spectrum LED curing light. The Vickers microhardness and the degree of conversion (DC) at the top and bottom surfaces were measured 24 h later before and after polishing the RBC. The microhardness measurements were made using 50, 100, 300 and 1000-gf loads with the same 8 s dwell time. The DC was measured in the same region using mid FT-IR spectroscopy. Repeated measures analysis of variance tests were used to determine if the surface (top vs. bottom) or the indenter loads had a significant effect on the VH, or if polishing affected the VH and the DC (alpha = 0.05). It was found that the indenter load did not affect the Vickers hardness of the unpolished top surface (*p* = 0.759), the polished top surface (*p* = 0.374), or the polished bottom surface (*p* = 0.083) of the Tetric EvoCeram Bulk Fill. Increasing the indenter load did increase the VH of the unpolished surface at the bottom (*p* < 0.0001). Polishing increased the DC by 13.5% at the top and by 46.7% at the bottom surface.

## 1. Introduction

With the worldwide agreement to phase down the use of amalgam [1] and the ban on using and exporting dental amalgam in the European Union (starting in January 2025) [2], the use of light-cured resin-based composites (RBCs) has become ubiquitous. Consequently, there has been much interest in determining the extent to which the RBC has achieved an “adequate” cure (polymerization) and how this is affected by the light curing unit (LCU), the type and shade of the RBC, and the thickness of the RBC. The extent of the polymerization that RBCs attain after light curing is often investigated by measuring its degree of conversion (DC) or surface hardness [3,4,5,6,7,8]. Despite the number of publications that have used hardness testing, a systematic review [9] of the depth of cure of bulk-fill resin composites concluded that there was significant methodological heterogeneity in the assessment methods and the outcomes of the studies that used hardness testing. This heterogeneity made it impossible to conduct a meta-analysis [9]. For example, this review found that some specimens were exposed to air for 24 h and were not polished before Knoop hardness testing [9,10]. Others were exposed to air for 24 h and were not polished before Vickers hardness testing [11,12,13,14,15,16]. One was not exposed to air and not polished before Vickers hardness testing [17]. Two were stored in water and not polished before Vickers hardness testing [18,19]. Several were polished before Knoop [20,21,22,23,24,25] or Vickers hardness testing and stored in air before hardness testing [26,27,28,29,30]. The studies also used a variety of indenter loads: 5000 gf [26], 1000 gf [15,30], 500 gf [11,13], 300 gf [14,17], 200 gf [12,28], 100 gf [21,25], 50 gf [16,24,31], and 10 to 50 gf [10,32].

### 1.1. Concerns When Using Hardness Tests: Effect of Indenter Load

Barcol, Brinell, Knoop, Vickers, and Martens hardness tests have all been used to measure the surface hardness of RBC, but RBC is often polished before testing [11,12,13,14,15,16,17,21,22,24,25,26,28,30,31,32,33,34,35,36,37,38,39,40,41,42]. Based on the hardness measurements made on the surface of the polished RBC specimens, there are proposals for percent values that deeper regions of the RBC should attain relative to that of a well-cured top surface. A value of 80% of the hardness at the top surface is often considered to be the borderline between sufficient and insufficient curing [17,20,43,44]. However, depending on the specific RBC, this value may be as low as 73% [45], or as high as 90% [46] of the maximum hardness for the RBC to be clinically successful.

Both the Vickers microhardness (HV) and Knoop microhardness (HK) tests are covered by the ASTM E384 standard [47]. To be classified as a microhardness test, the test force should be less than 1000-gf [47], and the indentation depth should be greater than 0.2 μm [48]. The difference between these two tests is related to the geometry of the indenter. After removing the indenter, the imprint left behind is a depression in the surface. The dimensions of these indents are measured and placed into the appropriate formula to provide units of hardness, and some also provide the indentation depth [49]. Without a flat, smooth surface, non-symmetrical and/or unreadable indentations will be made. Such indentations produce inaccurate indentations and inaccurate hardness values [48]. Consequently, in most studies, the surfaces of the RBCs are polished using copious water coolant to produce a flat surface before testing [20,21,22,23,24,25,26,27,28,29,30,45,50,51,52].

The indentation size effect (ISE) has been studied in dentistry [53,54,55,56,57] and has been reported to be significantly affected by the filler properties, indenter load, and loading frequency, but how the ISE affects hardness measurements is often overlooked. Indenter loads of 400-gf have been reported to increase the Knoop microhardness of unpolished RBCs, compared to when 200-gf is used [49]. However, this may be due to a measurement error since a lower indenter load will produce a smaller indent, and unless a larger magnification is used, it is more likely that the measurements made of the indentation will be imprecise [48,58]. Some researchers have used loads less than 50-gf [5,10,29,44,59], while others used loads of 1 kg and above [15,18,20,26,30,60,61], possibly to penetrate beyond the oxygen-inhibited layer that may be 11 to 23 μm thick, depending on the RBC tested [31]. However, in one study, when the surfaces of the contemporary RBCs were covered with an acetate strip before light curing, no resin-rich layers were found in any of the three unpolished RBCs [60].

### 1.2. Concerns When Using Hardness Tests: Effect of Polishing

The failure to recognize the potential impact of the indentation size, the lack of a standard test protocol for hardness evaluation, which indenter load to use, the magnification used when measuring the indents, the use of polished vs. unpolished samples, and the load time may be the underlying cause of the conflicting findings and conclusions among published studies that have used hardness testing [9]. Some believe that polishing is always required to remove any potential resin-rich surface layer on the RBC. Although polishing will remove any air-inhibited layer on the surface of the resin [31], cutting or polishing the RBC before hardness testing may alter the surface of the RBC and affect the results [60]. For example, when polishing the RBC specimens to obtain a flat surface before microhardness testing, the use of water coolant to prevent heat increase may preferentially remove the relatively hydrophilic free monomers, such as residual TEGDMA or other low molecular weight monomers that have some degree of water solubility [45,62,63] from the surfaces of the cut specimens. Some have also polished the RBC with an alcohol-based solution [7]. This will likely remove even more monomers and have an even greater effect than polishing with a water-based solution [64,65,66]. Thus, the effect of using different indenter loads, each producing different indentation depths, should be investigated to see if increasing the indenter load will influence the microhardness value of the RBC.

The null hypotheses tested were as follows:

1. Polishing the surface of the RBC would not affect the Vickers microhardness values;
2. The indenter load would not affect the Vickers microhardness values obtained from the unpolished and polished RBC surfaces;
3. Polishing the surface of the RBC would not affect the DC.

## 2. Methods

### 2.1. Sample Preparation

To investigate the indentation size effect when using different indenter loads and the effect of polishing to produce a flat surface, the microhardness of the top and bottom surfaces of one resin-based, bulk-filled composite were measured. To reduce the number of variables, only one RBC (Lot# V23428, Tetric EvoCeram Bulk Fill, shade IVA, Ivoclar, Schaan, Liechtenstein) was used. Tetric EvoCeram Bulk Fill is a light-cured, nano-hybrid composite that can be photo-polymerized in 10 s when it receives more than 1000 mW/cm^2^. This RBC has an overall filler content of approximately 61% (vol.) and 17% ‘Isofillers’ [67].

One LCU and one standard loading time (8 s) were used. However, the indents were measured using different magnifications (5×, 10× or 20×, depending on their size). Five RBC samples were prepared in 4 mm thick metal rings having 12 mm wide inner diameter holes_._ Thin spacers of polyester sheets that were 0.25 mm thick were placed on the top and bottom edges of the ring to allow for a controlled amount of RBC that could be polished away. Since the background behind the RBC can affect the depth of cure [68], the molds were placed on a polyester strip over a flat piece of previously cured RBC (Filtek Supreme Ultra shade A2B, 3M Oral Care, St. Paul, MN, USA). This provided a controlled background for the RBC that would be similar to the clinical conditions. The uncured composite was packed into the mold, slightly over-filling it. Another polyester strip was placed on the top, and the whole assembly was pressed flat, using a glass mixing slab, to create a surface that was flat and parallel with respect to the top and bottom of the sample. This process also allowed the excess composite material to escape. The specimens were light cured for 10 s at a 0 mm distance from the surface of the sample using a Polywave^®^ light emitting diode curing light (Bluephase^®^ Style, Ivoclar Vivadent), with the tip perpendicular to the mold surface and directly in contact with the polyester strip. This broad spectrum Polywave^®^ curing light was used to match the brand of curing light to the RBC, which contains both camphorquinone and Ivocerin^®^ photoinitiators [67]. After exposure to the curing light, the molds were stored for 24 h at 37 °C in the dark. Based on a pilot study and previous research [3,69], this time was sufficient to reach stable post-irradiation hardness and DC values. The polyester strips were only removed from the top and bottom surfaces 24 h after the RBC specimens were made, and the samples were allowed to return to room temperature (~22 °C) before hardness testing.

### 2.2. Radiant Power, Radiant Emittance, Radiant Exposure, and Emission Spectrum

To determine the radiant power, radiant emittance (irradiance at the light tip), radiant exposure, and emission spectrum received by the RBC, the total light output was measured using a 6-inch integrating sphere (Labsphere, North Sutton, NH, USA) connected to a fiber-optic spectrometer (USB 4000, Ocean Optics, Dunedin, FL, USA). The light output was also measured through a 1 mm^2^ aperture into the same sphere. This measurement was not the total output emitted from the LCU tip end, but it was where the Vickers hardness and the degree of conversion values were later measured. Software (OceanView, Ocean Optics) was used to collect and analyze the data. The light beam profiles were evaluated with a Laser Beam Profiler. This device uses a digital camera with a 50 mm focal distance lens (USB-L070, Ophir Spiricon, Logan, UT, USA) that was positioned at a fixed distance from a 60-degree holographic screen (Thor Laboratories, Newton, NJ, USA). The distribution of the emitted radiant power from the tip of the LCU, when it was positioned almost in contact with the holographic screen, was recorded using BeamGage v.6.6 software (Ophir Spiricon). The effect of the ambient light was eliminated by normalizing every pixel to a similar level using UltraCal (Ophir Spiricon) before each measurement.

### 2.3. Degree of Conversion (DC) Measurements

At the same time that the samples were made for Vickers microhardness measurements, five additional specimens were prepared under identical conditions to measure the DC at the top and the bottom surfaces of the RBC before and after polishing. After exposure to the curing light, the molds were stored for 24 h at 37 °C in the dark before the DC at the top and bottom specimen surfaces were measured using Attenuated Total Reflectance (ATR) Fourier-transform (FT-IR) mid-infrared spectroscopy (Vertex 70, Bruker, Billerica, MA, USA). To measure the DC, a baseline correction was performed using software (Opus v 7.2, Bruker). This process monitored the decrease in the integrated area of an aliphatic C=C peak at 1638 cm^−1^ relative to an internal reference aromatic C=C peak at 1608 cm^−1^. The static FT-IR spectra were obtained at a resolution of 4 cm^−1^, and the DC was calculated from an average of 30 scans. The mid-FT-IR instrument had been previously calibrated based on a technique reported by Rueggeberg et al. [70] and shown to be a second-order polynomial (y = 0.141x^2^ + 1.1424x) with a degree of correlation (R^2^) of 1. This correction was included in the following DC calculation (1).

(1) $$\;DC=100\%\times \left[1-\frac{{0.141\left(\frac{{Abs}_{Aliphatic}}{{Abs}_{Aromatic}}\right)}_{Polymer}^{2}+{1.1424\left(\frac{{Abs}_{Aliphatic}}{{Abs}_{Aromatic}}\right)}_{Polymer}}{{0.141\left(\frac{{Abs}_{Aliphatic}}{{Abs}_{Aromatic}}\right)}_{Monomer}^{2}+{1.1424\left(\frac{{Abs}_{Aliphatic}}{{Abs}_{Aromatic}}\right)}_{Monomer}}\right]$$

### 2.4. Polishing

After initial monomer conversion and microhardness measurements had been made on both surfaces of the RBCs, the samples were hand polished on a rotary polishing device using copious water coolant. The outer shape of the test rings was asymmetrical, allowing them to be reproducibly positioned into a jig in the hardness tester with high precision. The first stage of polishing used an abrasive paper (P800C, Klingspor, Haiger, Germany) to remove most of the RBC material. Next, a slurry containing 3 μm alumina particles (Buelher, Lake Bluff, IL, USA) in water was used on a rotating cloth disk for 5 min. This process was followed by final polishing using a slurry of 0.3 μm alumina particles (Buelher) in water on a separate cloth sheet for an additional 5 min to produce a mirror-like flat surface finish suitable for microhardness testing. After polishing, and within 6 h of the initial pre-polish measurements, a second round of microhardness and DC measurements were obtained on both the top and bottom surfaces using the same test conditions and at similar locations to those made before polishing.

### 2.5. Vickers Microhardness (VH) Measurements

**Figure 1:**
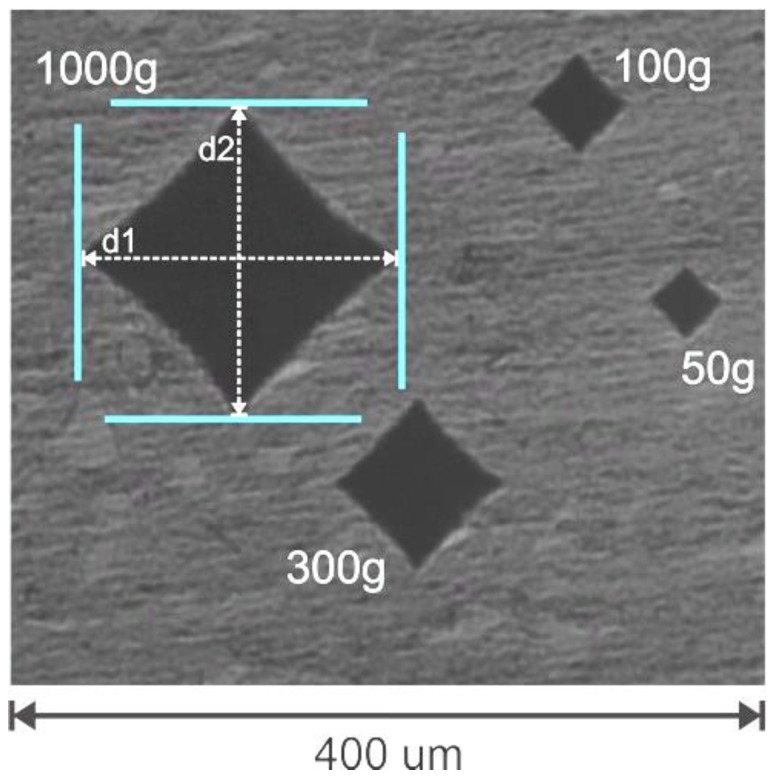
Examples of the four Vickers indentations using the four different loads, but the same load time. The lines in the 1000 gf indent show where the Vickers microhardness measurements d1 and d2 were made on each indent. (Note: For photographic purposes, the indentations are closer than in the study).

Vickers microhardness measurements were made using a microhardness tester (HM 123, Mitutoyo, Kawasaki, Kanagawa, Japan). Indentations were made at similar locations across the specimens using 50-gf, 100-gf, 300-gf, and 1000-gf loads (Figure 1). The hardness tester was calibrated to produce identical hardness results using 5×, 10× and 20× magnification. To determine the ISE, all the indents were made using the same 8 s dwell time so that different indent sizes were made. The indents were made at 5 locations in the center of the specimen (close to where the DC was also measured) at both the top and bottom surfaces of each sample. The indents were spaced 1 mm apart (Figure 1) such that the space between the indents was at least five times the width of the indent and in the region where the initial hardness and DC measurements had been made. Depending on the size of the indent, the indent was measured using 5× (1000 gf), 10× (300 and 100 gf), or 20× (50 gf) magnification so that the images on the screen were similar in size.

In total, 400 Vickers hardness measurements were made (5 indents × 4 loads × 2 surface locations × 2 surface conditions × 5 repeats).

### 2.6. Statistical Analyses

A repeated measurements analysis of variance (The R Foundation for Statistical Computing. R version 3.4.2, https://www.r-project.org) was used to test whether the factors of surface (top vs. bottom), load and condition (polished vs. unpolished) had a significant effect on the Vickers microhardness or the degree of conversion (alpha = 0.05). Given that there were five specimens and five measurements for each combination of factors per sample, the average of the five VH measurements was used for the analysis, i.e., 80 observations for hardness = (4 loads × 2 conditions × 2 surfaces × 5 samples), and 20 observations for the degree of conversion = (5 samples × 2 conditions × 2 surfaces).

## 3. Results

**Figure 2:**
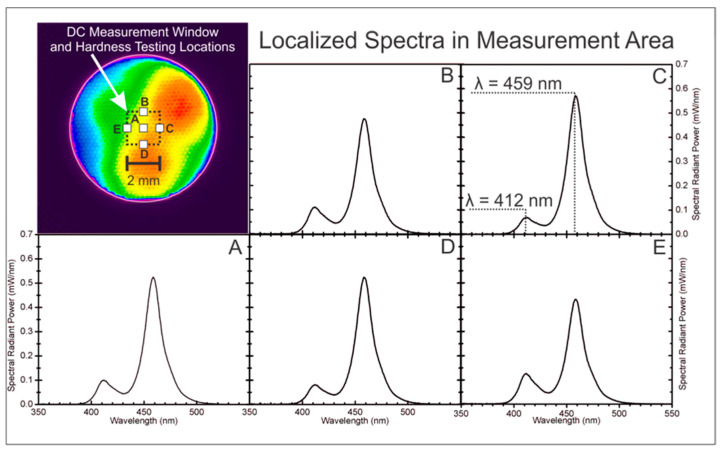
Beam profile and the emission spectra from the LCU. The DC was measured at location (**A**). The Vickers microhardness was measured at five locations: (**A**–**E**). The light output where the measurements were made was homogeneous.

The Bluephase^®^ Style delivered a total radiant power of 800 ± 6 mW, a radiant emittance (irradiance) of 1345 ± 10 mW/cm^2^, and a radiant exposure of 13.5 ± 0.1 J/cm^2^ to the top surface of the specimens. Although there was some inhomogeneity in the irradiance distribution across the entire light tip, all five locations where the specimens were measured for microhardness and DC received similar wavelengths of light. This was confirmed by the measurements made through a 1 mm^2^ aperture into the integrating sphere (Figure 2).

### 3.1. Indentation Depth

When using the same 8 s dwell time, the indentation depth into the RBC ranged from 5.3 μm (50-gf) to 26.1 μm (1000-gf), as seen in Table 1. This depth was less than the amount of RBC removed by polishing (approximately 250 μm).

### 3.2. Effect of Polishing on Vickers Microhardness

**Figure 3:**
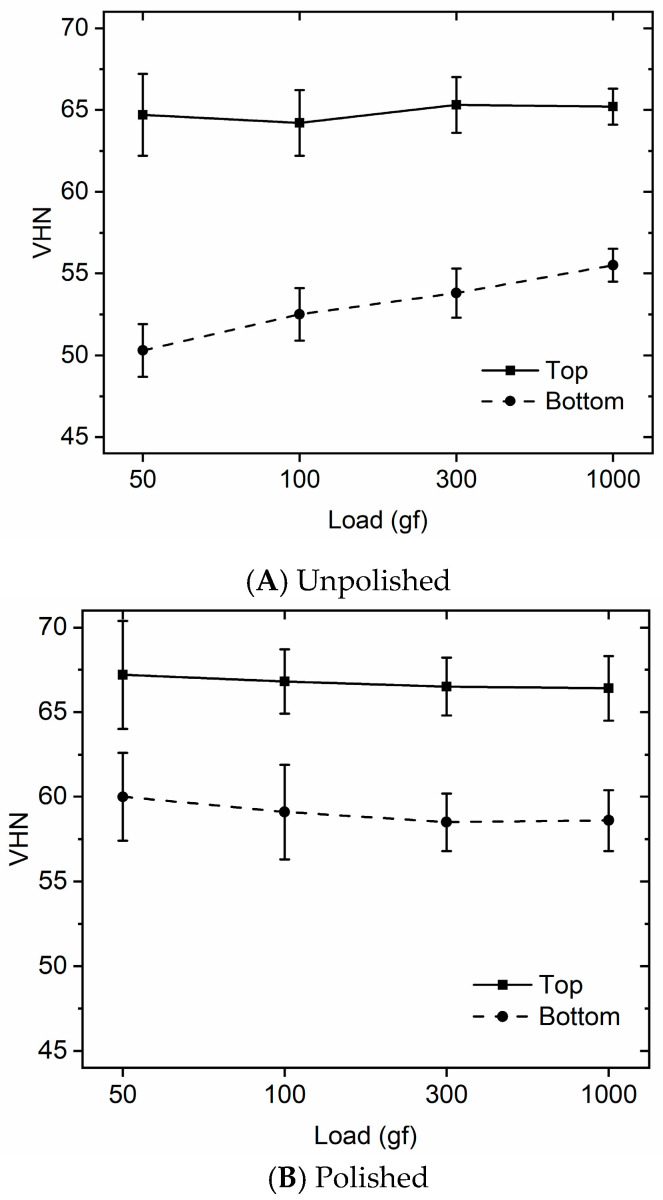
Effect of indenter load at the top and bottom surfaces of the unpolished (**A**) and polished (**B**) specimens. Note the different effects between the top and bottom surfaces (n = 5).

Depending on the surface and indenter load, the VH of the polished RBCs ranged between 58.5 ± 1.7 and 67.2 ± 3.2 (Table 2). The bottom-to-top surface ratios ranged from 88.0% to 89.3 %, depending on the load used. As illustrated in Figure 3A,B, the three-way interaction of the condition, the surface, and the load was statistically significant at the 5 % level (*p* = 0.037), mainly because the unpolished surface at the bottom surface had a much lower hardness value before polishing. The two-way interaction of the condition and the surface was also highly significant (*p*-value < 0.0001), indicating that polishing increased the microhardness values at the top and the bottom surface. The indenter load had no significant effect on the polished specimens at the top surface (*p* = 0.374), the polished bottom surface (*p* = 0.083), or the unpolished top surface (*p* = 0.759). However, using different loads had a significant positive effect on the unpolished bottom surfaces (*p* < 0.0001). At the unpolished bottom surfaces, a load of 50-gf produced a hardness value that was significantly lower than when using loads of 100, 300, and 1000-gf, at *p*-values of 0.02, 0.01, and 0.01, respectively. The Vickers microhardness obtained at the bottom surfaces using the 100-gf load was significantly lower than that measured when using 1000-gf at *p* = 0.01, but not at *p* = 0.05. Using the 300-gf load did not produce significantly lower VH values compared to when using 1000-gf (*p* > 0.05).

### 3.3. Effect of Polishing on DC

Table 3 shows that polishing produced a statistically significant 13.5% increase in the DC values at the top. There was an even greater increase of 46.7% at the bottom surface when the specimens were polished, compared to the unpolished surface (*p* < 0.0001).

## 4. Discussion

Polishing the RBC consistently increased the microhardness values of the top and the bottom surfaces (*p*-value < 0.0001). Thus, the first null hypothesis, that polishing would not significantly affect the microhardness values, was rejected. At the bottom, on the unpolished surfaces, a load of 50-gf produced a lower Vickers hardness number than when using 100, 300, and 1000-gf loads (*p*-values of 0.02, 0.01, and 0.01, respectively). Although the effect of different loads was not significant on the top polished surface (*p* = 0.374), the bottom polished surface (*p* = 0.083), or on the unpolished top surface (*p* = 0.759), the use of different loads did have a significant effect on the unpolished bottom surface (*p* < 0.0001). Thus, the second null hypothesis, that the indenter load would have no effect, was only partially accepted. The third null hypothesis, that polishing would not affect the DC, was rejected because polishing produced a large, significant 13.5% increase (*p* < 0.0001) in the DC values at the top and an even more substantial increase of 46.7% at the bottom surface (Table 3).

When exposed for 10s to the Bluephase^®^ Style Polywave^®^ curing light, the bottom–top Vickers microhardness ratio of the 4 mm thickness of the unpolished Tetric Evoceram Bulk Fill was above 80% when loads of 100-gf and above were used. This ratio increased to approximately 88.5% after the RBC was polished. Since a value of 80% of the hardness at the top surface is often considered as the borderline between sufficient and insufficient curing [17,20,43,44], achieving an 88.5% bottom–top hardness ratio suggests that Tetric Evoceram Bulk Fill can be adequately light cured to a depth of 4 mm in 10s. Since it is now known that polishing increases the DC, the 88.5% bottom–top hardness ratio achieved after polishing must now be questioned. However, the before-polishing value ranges of 81.8% at 100-gf and 85.1% at 1000-gf were all still above the minimum 80% threshold.

Even though different magnifications were used to account for the different sizes of the indents, the results of this study highlight the potential effect of the indentation size (ISE). Dental RBCs are composed of a polymer matrix and various inorganic fillers. Under lower indentation loads, the hardness measured can be dominated by the properties of the individual filler particles. However, as the load increases and the depth of the indent increases, differences in the filler size and shape can affect the ISE. Smaller, spherical fillers create a different distribution of stress and deformation around the indenter than larger or irregularly shaped fillers. This affects the hardness measurements differently depending on the load and the depth of the indent [55]. Larger fillers may produce a stiffer resistance at shallower indentations, while irregular fillers can create micro-stress concentrations that alter the hardness readings as the depth increases. The ISE is also affected by the viscoelastic behavior of the resin matrix. At shallow depths and lower loads, the resin may respond elastically. However, as the depth increases, the resin matrix deforms more plastically, and these effects will affect the hardness measurement. As illustrated in Table 2 and Table 3, polishing the top surface had a smaller positive impact on the microhardness and the DC. This result likely occurred because the RBC at the top surface was already well polymerized. Consequently, polishing had little effect. In contrast, polishing increased the values obtained at the bottom surface, where the RBC was less well polymerized. The beneficial effects of polishing on the observed properties of the RBC are supported by other investigations [69,71] that also verify the beneficial effects of polishing on a minifilled RBC, and by studies on the mechanochemical effects of polishing [72,73].

The results of this study support the previous concerns raised about how the conclusions derived from reporting the hardness and monomer conversion values of polished samples of RBCs are likely to have been artificially increased [10,74]. Sequential polishing removes the plastically abrasion-deformed layer, which has been reported to be approximately 1 μm thick [60] and hence would have negligible effects when loads of 100-gf and above are used (Table 1). As in other studies, copious water was used in this study as a coolant to reduce any heat-related issues during polishing. However, water has been reported to have a significant dissolving effect when low (4 J/cm^2^) radiant exposures were delivered [45] because one of the most abundant monomers that is left unpolymerized in the RBC is TEGDMA, and this monomer is partially water soluble. In contrast, the poor aqueous solubility of BisGMA will limit its release from the polished interface while its strong intermolecular hydrogen bonding provides mechanical reinforcement, even if present as the free monomer, which is not the case for more hydrophilic monomers such as TEGDMA [62]. Thus, a likely explanation of the perceived enhanced degree of conversion after polishing the less well-polymerized bottom surfaces comes from the localized selective extraction of hydrophilic monomers that not only directly enhance conversion but also artificially inflate it to even higher values due to the normalization relative to the aromatic internal reference peak during the €s. Any local loss of TEGDMA that had been washed away in the water coolant would appear as a loss of aliphatic C=C bonds. This would artificially raise the DC calculation when the uncured RBC is used as the internal reference. As shown in Table 3, this effect would be greater in areas of lower conversion where there is more of the free monomer to wash away and a lower network density where the sol fraction at ~45% DC would be about 2x that of the same network at 70% conversion [63].

The Vickers indenter is a symmetrical, four-sided pyramid. Both long sides of the base of the pyramid indentation on the specimen surface, as shown in Figure 1, are measured and entered into a formula to calculate the surface hardness. Thus, other RBCs that have Vickers hardness values of approximately 60 would also be expected to have similar indentation depths into the RBC of at least 5.3 μm (50-gf) and up to 25.4 μm (1000-gf) when using the same 8 s dwell time, as seen in Table 1. This 25.4 μm depth matched the 20 to 35 μm depth reported in a previous study that also used a 1000-gf load and the Vickers test [60].

The ISE was the greatest for the lowest load and the smallest indent. The 50-gf load produced the shallowest indent (5.3–6.1 μm), and the 50-gf Vickers results were the most affected by polishing. Although 1000-gf has been recommended to be the minimum indentation load level to measure the bulk material response of dental composite materials [60] and is used by some [15,30], a 1000-gf load is the highest load allowed in the ASTM E384 microhardness test [47] and it may deform the specimen. Since the present study found no difference between all four loads when used to measure the polished RBC surfaces (Table 2, Figure 3), it is proposed that the 300-gf load used by many researchers [14,17,75,76,77] is a reasonable compromise between achieving an adequate indent depth and not deforming the RBC specimen. This load is high enough to penetrate (~13 μm) into the RBC and this is beyond the air inhibited surface layer.

Although polishing the RBC is customary intra-orally, only the occlusal surface of the restorations is polished, leaving the bottom and sides of the RBC restoration untouched. The reader should recognize that polishing improves the hardness and the DC of the RBC. Polishing also has a greater impact on RBC surfaces that are less well-polymerized (Figure 3), obfuscating the ability to discern real differences in the surface properties of the RBC produced by different light curing conditions. Consequently, when reading and interpreting research results, the reader should consider the methods used to obtain the data and what those data represent. What was the indenter load, what was the dwell time, what was the depth of the indent, what magnification was used to read the indent, and were the specimens polished?

### Limitations

This study only used one RBC. Since different resins and fillers are used in other brands of RBC, further studies should be conducted to determine how the choice of RBC, the loading time or the hardness tests (such as Knoop or Martens) is related to the indentation size effect. Further studies should also be conducted to examine if the loss of TEGDMA and other water-soluble monomers during polishing is causing the increase in the DC. Detailed high-performance liquid chromatography (HPLC) analysis of the eluates from water-cooled polishing wheels would help confirm that suspicion. The effect of using a water-based polishing paste compared to an alcohol-based polishing paste used by some [7] should also be investigated. The ATR FT-IR test method only penetrates a few microns (~2 μm) beneath the surface; thus, it is just the polished surface being measured. Transmission FT-IR may be less affected by polishing since this is an average measurement of the DC through the RBC rather than the DC at the surface. Additionally, near IR measurements that base their DC calculations on the changes in a single peak that are not associated with a soluble monomer may be less affected by polishing.

## 5. Conclusions

Within the limitations of this study, the following conclusions were made:

1. When using the Vickers microhardness test, polishing increased the microhardness values at the top and the bottom surfaces (*p*-value < 0.0001);
2. The indenter load had no significant effect on the Vickers hardness measured on the unpolished top surface (*p* = 0.759), the polished top (*p* = 0.374), and bottom surfaces (*p* = 0.083) of the Tetric EvoCeram Bulk Fill. However, increasing the indenter load did increase the VHN of the unpolished bottom surface (*p* < 0.0001);
3. Polishing significantly increased the DC by 13.5% at the top and by 46.7% at the bottom surface (*p* < 0.0001);
4. When exposed for 10s to the Bluephase Style Polywave^®^ curing light, the bottom–top Vickers microhardness ratio of the 4 mm thickness of the polished Tetric Evoceram Bulk Fill was approximately 88.5%;
5. A 300-gf load should be high enough to penetrate beyond any air-inhibited surface layer and is a reasonable compromise between achieving an adequate indent depth and not deforming the RBC specimen.

## Figures and Tables

**Table 1:** Mean indentation depth (um) ± standard deviation (S.D.) using the four using four different loads (50, 100, 300 and 1000-gf).

SURFACE	TREATMENT	MEAN INDENTATION DEPTH (microns) (S.D.)							
		INDENTER LOAD (gf)							
		50		100		300		1000	
TOP	UNPOLISHED	5.4	(0.1)	7.7	(0.1)	13.2	(0.2)	24.1	(0.2)
	POLISHED	5.3	(0.1)	7.5	(0.1)	13.1	(0.2)	23.9	(0.3)
BOTTOM	UNPOLISHED	6.1	(0.1)	8.5	(0.1)	14.5	(0.2)	26.1	(0.2)
	POLISHED	5.6	(0.1)	8.0	(0.2)	13.9	(0.2)	25.4	(0.4)

**Table 2:** Mean Vickers microhardness number (VHN) ± standard deviation (S.D.) at the top and bottom surfaces measured using four different loads (50, 100, 300 and 1000-gf) both before and after polishing.

Surface	Mean VHN (S.D.)								
	Indenter Load (gf)								Within Row Comparison
	50		100		300		1000		*p*-Value
Top: unpolished	64.7	(2.5)	64.2	(2.0)	65.3	(1.7)	65.2	(1.1)	0.759
Bottom: unpolished	50.3	(1.6)	52.5 ^A^	(1.6)	53.8 ^A,B^	(1.5)	55.5 ^B^	(1.0)	<0.0001
Bottom–top ratio	77.7		81.8		82.4		85.1		
	50		100		300		1000		*p*-value
Top: polished	67.2	(3.2)	66.8	(1.9)	66.5	(1.7)	66.4	(1.9)	0.374
Bottom: polished	60.0	(2.6)	59.1	(2.8)	58.5	(1.7)	58.6	(1.8)	0.083
Bottom–top ratio	89.3		88.5		88.0		88.3		

Indenter load had no significant effect on the polished and the unpolished top surfaces and the polished lower surfaces (*p* > 0.05). The superscript letters ^A^ and ^B^ denote values at the bottom of the unpolished surfaces that were not significantly different (*p* > 0.05).

**Table 3:** Effect of polishing on the degree of conversion (DC) ± standard deviation (S.D.) at the top and bottom surfaces.

SURFACE	TREATMENT	% DC (S.D.)		% Increase
TOP	UNPOLISHED	59.9	(2.7)	
	POLISHED	68.0 *	(2.7)	13.5
BOTTOM	UNPOLISHED	44.3	(3.6)	
	POLISHED	65.0 *	(3.7)	46.7

Within each surface, the DC values * were significantly greater after polishing (*p* < 0.0001).

## Data Availability

The original contributions presented in this study are included in the article. Further inquiries can be directed to the corresponding author(s).
